# Theoretical Analysis for Bending of Single-Stranded DNA Adsorption on Microcantilever Sensors

**DOI:** 10.3390/s18092812

**Published:** 2018-08-26

**Authors:** Zou-Qing Tan, Yang-Chun Chen, Neng-Hui Zhang

**Affiliations:** 1 School of Mechanical Engineering, Changzhou University, Changzhou 213164, China; 17000085@smail.cczu.edu.cn; 2 Shanghai Institute of Applied Mathematics and Mechanics, Shanghai University, Shanghai 200072, China; nhzhang@shu.edu.cn; 3 Department of Mechanics, College of Sciences, Shanghai University, Shanghai 200444, China

**Keywords:** biosensor, microcantilever, DNA adsorption, mean-field theory, energy method

## Abstract

An energy-based model is presented to establish the bending deformation of microcantilever beams induced by single-stranded DNA (ssDNA) adsorption. The total free energy of the DNA-microcantilever sensor was obtained by considering the excluded-volume energy and the polymer stretching energy of DNA chains from mean-field theory, and the mechanical energy of three non-biological layers. The radius of curvature and deflection of the cantilever were determined through the minimum principle of energy. The efficiency of the present model was confirmed through comparison with experimental data. The effects of length, grafting density, salt concentration, thickness, and elastic modulus of substrate on tip deflections are also discussed in this paper. These factors can significantly affect the deflections of the biosensor. This work demonstrates that it is useful to develop a theoretical model for the label-free nanomechanical detection technique.

## 1. Introduction

Microcantilever-based biosensors have attracted a great deal of attention due to their small size, fast response, high sensitivity, low cost, and suitability for parallelization into arrays [1,2,3]. The label-free microcantilever-based biosensors have been used in the detection of small molecules [4], microcystin–leucine–arginine [5], DNA hybridization [6], proteins–nucleic acids binding [7], BRAF mutation in RNA from melanoma cells [8], water–DNA interactions [9,10], and human papilloma virus infections [11].

When single-stranded DNA (ssDNA) adsorption occurs on one side of a cantilever in a solution, a mechanical bending of the cantilever occurs due to the change in surface stresses, thereby translating biochemical interactions into a nanomechanical response. Microcantilever-based biosensors have two operation modes: static bending mode and dynamic frequency mode. Compared to static detection, real-time dynamic detection is more challenging because the sensors are usually immersed in a liquid environment. Recently, curvature measurements have been one of the most common techniques for measuring stresses in film determined by DNA adsorption [12]. Many experiments have shown that the deflections of a DNA-microcantilever sensor can be induced by three main types of factors: (1) properties of the DNA chain, such as length, sequence, and grafting density [13]; (2) properties of substrate, such as geometry, material, and shape [14,15]; (3) environmental changes, such as moisture concentration [9], buffer salt solution concentration [16], and temperature [17]. However, the molecular mechanism involved in adsorption-induced stress is not completely understood [12,18].

The challenge in theoretically modeling such a complex system and developing the design rules is that the DNA-microcantilever sensor is essentially a kind of biochemical-mechanical coupling system [19]. The stress in the DNA film causes the bending of the cantilever. In return, DNA interactions are modified by the bending cantilever. Recently, some methods were established to understand this mechanical behavior. Based on a classical macroscopic piezoelectric theory, Zhang et al. [20] developed a phenomenological model to interpret the bending of the cantilever. According to Daoud and Cotton’s blob model for cylindrical polymer brushes; Hagan et al. [21] presented a simplified two-layered model to study the influence of conformational entropy on such motion. Subsequently, Tan and Zhang expanded upon this method by considering the effects of coating thin layers on the mechanical energy of biosensors [22]. Utz and Begley [23] established a relationship between molecular properties and adsorption-induced stresses in polymer brushes using the thermal blob. However, the deflections from most of the abovementioned theories appear to be smaller than the related experimental data. In this paper, we will present an alternative model for the nanomechanics of microcantilevers induced by ssDNA adsorption.

This paper develops an energy-based method for the nanomechanics of ssDNA adsorption on microcantilever sensors. The free energy of ssDNA film was obtained through mean-field theory. The mechanical energy of three non-biological layers was considered. The radius of curvature and deflection of the DNA-microcantilever sensor were obtained through the principle of minimum energy. The deflections predicted by the present model were compared with experimental data. The effects of DNA film properties (including chain length, grafting density, and salt concentration) and substrate properties (including thickness, length, and elastic modulus) on deflections are also discussed.

## 2. Theory and Modeling

**Figure 1:**
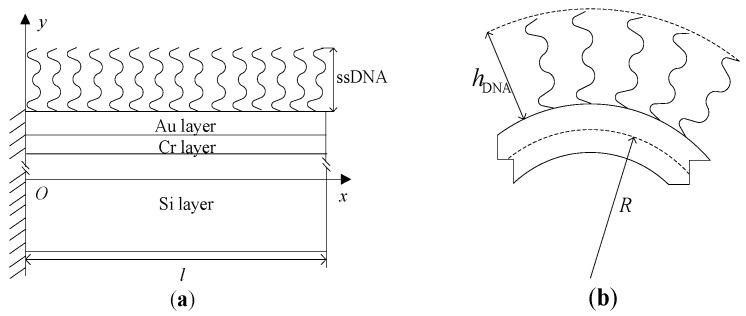
Schematic showing a DNA-microcantilever sensor and its coordinate system: (**a**) before deformation; (**b**) after deformation.

The DNA-microcantilever sensor is a multilayer structure, and is shown in Figure 1, which consists of two parts: DNA film and non-biological layers. The non-biological layers include three layers [24]. Each of these non-biological layers has its own function. The silicon is used as the substrate structural material of the cantilever. A thin gold film is deposited and patterned on one side of the cantilevers to allow immobilization of biomolecules through gold-thiol (Au-S) bonds. For good adhesion between gold and silicon, a thin chrome film is deposited on the silicon cantilever before the deposition of gold. For simplicity, the non-biological layers are perfectly elastic and homogeneous. Assuming that the interface bonding between different layers is perfect, the strain on the surface of two adjacent layers must be equal during the bending of the cantilever. In addition, the ordered conformation of thiolated ssDNA molecules on gold surfaces is assumed. The centroidal axis of the cantilever is taken as the coordinate axis *x*; its vertical direction along the cantilever thickness is consistent with that of the coordinate axis *y*. Here, *l*, *b* and *h* are the length, width and thickness, respectively; *h*_Au_, *h*_Cr_, and *h*_Si_ represent the respective thicknesses, and *h = h*_Au_ + *h*_Cr_ + *h*_Si_; *E*_Au_, *E*_Cr_, and *E*_Si_ are the respective elastic moduli.

The total free energy of the DNA-microcantilever sensor can be written as:

(1) $$U_{\mathrm{tot}}=U_{e}+U_{s}+U_{m}$$

where *U*_e_, *U*_s_, and *U*_m_ are the excluded-volume energy, the polymer stretching energy, and the mechanical energy of non-biological layers, respectively. *U*_e_ + *U*_s_ represents the contribution from the conformational entropy of the ssDNA chain.

According to a Flory-type mean-field argument for the excluded-volume energy per DNA chain [25]:

(2) $$U_{e}=\frac{k_{B}TvN^{2}}{d^{2}h_{\mathrm{DNA}}\left[1+\frac{h_{\mathrm{DNA}}}{2(R+h/2)}\right]}$$

where *R* is the radius of curvature of the shape adopted by the cantilever; *h*_DNA_ is the thickness of the DNA film; *N* is the number of nucleotides per DNA chain; $d\approx 1/\sqrt{\eta }$ is the grafting distance of the DNA chain, where η is the grafting density of the DNA chain; *k*_B_ is the Boltzmann constant; *T* is the absolute temperature; $v=l_{s}^{2}\kappa^{-1}$ is the excluded-volume parameter of a Kuhn segment, where the unit of the equivalent freely-jointed chain is called a Kuhn segment; $\kappa^{-1}$ is the local Debye length (≈0.3 nm/$\sqrt{I}$), where *I* denotes the local salt concentration; *l*_s_ (=1.5 nm) is the statistical segment length [21].

Because the max deflections of the biosensor due to DNA adsorption are less than 10% of the beam length, small deflections of cantilever beams are considered. For a small deflection, the radius of curvature of the beam is much larger than the thickness in this model, that is, *R* >> *h*, from Equation (2). An approximate expression of the excluded-volume energy per DNA chain can be written as:

(3) $$U_{e}=\frac{k_{B}TvN^{2}}{d^{2}h_{\mathrm{DNA}}\left(1+\frac{h_{\mathrm{DNA}}}{2R}\right)}$$

The polymer stretching energy of a Gaussian chain is written as [25]:

(4) $$U_{s}=\frac{3k_{B}Th_{\mathrm{DNA}}^{2}}{2a^{2}N}$$

where *a* is the length of *a* Kuhn segment.

Because the beam deflection readout of each cantilever has an accuracy of 0.1 nm [26], the influence of coating thin layers on the deflection cannot be neglected [27]. Therefore, in order to describe the deformation more accurately, according to the linear elastic theory, the mechanical energy of three non-biological layers (Si–Cr–Au) can be obtained as:

(5) $$U_{m}=\frac{{\left(EI\right)}_{\mathrm{tot}}}{2}{\left(\frac{d}{R}\right)}^{2}$$

where (*EI*)_tot_ is the total bending stiffness for multilayer beams, and it can be used as a substitute for the bending stiffness of layers as follows [28]:

(6) $${\left(EI\right)}_{\mathrm{tot}}=2n_{1}-\frac{n_{2}^{2}}{2n_{3}}$$

In which $n_{1}=\frac{b}{24}[E_{\mathrm{Au}}h_{\mathrm{Au}}(3h^{2}-6hh_{\mathrm{Au}}+4h_{\mathrm{Au}}^{2})+E_{\mathrm{Si}}h_{\mathrm{Si}}(3h^{2}-6hh_{\mathrm{Si}}+4h_{\mathrm{Si}}^{2})+E_{\mathrm{Cr}}(h^{3}-3h^{2}h_{\mathrm{Au}}+6hh_{\mathrm{Au}}^{2}-4h_{\mathrm{Au}}^{3}-3h^{2}h_{\mathrm{Si}}+6hh_{\mathrm{Si}}^{2}-4h_{\mathrm{Si}}^{3})],$ $n_{2}=\frac{b}{2}\left[E_{\mathrm{Au}}h_{\mathrm{Au}}(h-h_{\mathrm{Au}})+E_{\mathrm{Si}}h_{\mathrm{Si}}(h_{\mathrm{Si}}-h)+E_{\mathrm{Cr}}h_{\mathrm{Cr}}(h_{\mathrm{Si}}-h_{\mathrm{Au}})\right],\;n_{3}=\frac{b}{2}(E_{\mathrm{Au}}h_{\mathrm{Au}}+E_{\mathrm{Cr}}h_{\mathrm{Cr}}+E_{\mathrm{Si}}h_{\mathrm{Si}})$.

The dimensionless scaling variable α and dimensionless grafting density β is introduced as follows:

(7) $$\alpha =\frac{h_{\mathrm{DNA}}}{2R},\;\beta ={\left(\frac{d}{aN^{\mu }}\right)}^{-2}$$

where μ (=0.6) is the Flory exponent [22,23].

Substituting Equations (3)–(5) into Equation (1) and using the dimensionless variables α and β, the total free energy of the DNA-microcantilever sensor can be written as:

(8) $$U_{\mathrm{tot}}=\frac{v\beta N^{4/5}k_{B}T}{a^{2}h_{\mathrm{DNA}}(1+\alpha )}+\frac{3h_{\mathrm{DNA}}^{2}k_{B}T}{2a^{2}N}+\frac{2{\left(EI\right)}_{\mathrm{tot}}{\left(aN^{3/5}\alpha \right)}^{2}}{\beta h_{\mathrm{DNA}}^{2}}$$

By minimizing the total free energy with respect to α and *h*_DNA_, $\partial U_{\mathrm{tot}}/\partial \alpha =0$ and $\partial U_{\mathrm{tot}}/\partial h_{\mathrm{DNA}}=0$, we can yield the following equations:

(9) $$\frac{v\beta N^{4/5}k_{B}T}{a^{2}h_{\mathrm{DNA}}{\left(1+\alpha \right)}^{2}}-\frac{4{\left(EI\right)}_{\mathrm{tot}}{\left(aN^{3/5}\right)}^{2}\alpha }{\beta h_{\mathrm{DNA}}^{2}}=0$$

(10) $$\frac{v\beta N^{4/5.}k_{B}T}{a^{2}h_{\mathrm{DNA}}^{2}(1+\alpha )}-\frac{3h_{\mathrm{DNA}}k_{B}T}{a^{2}N}+\frac{4{\left(EI\right)}_{\mathrm{tot}}{\left(aN^{3/5}\alpha \right)}^{2}}{\beta h_{\mathrm{DNA}}^{3}}=0$$

Solving Equation (9), the thickness of the DNA film can be obtained as:

(11) $$h_{\mathrm{DNA}}=\frac{4{\left(EI\right)}_{\mathrm{tot}}a^{4}N^{2/5}}{k_{B}Tv\beta^{2}}\alpha {\left(\alpha +1\right)}^{2}$$

By combination of Equations (10) and (11), the radius of curvature of the DNA-microcantilever sensor satisfies the following equation:

(12) $${\left(1+\alpha \right)}^{8}\alpha^{3}-\gamma^{3}(2\alpha +1)=0$$

where $\gamma =\frac{k_{B}T}{4\sqrt[{3}]{3}{\left(EI\right)}_{\mathrm{tot}}}{\left(\frac{v^{1/3}}{a}\right)}^{4}N^{1/5}\beta^{7/3}$.

Solving Equation (12) and substituting it into Equation (7) yields the radius of curvature. Using the Euler–Bernoulli beam theory for a small deflection, the deflection of the cantilever is predicted by [27]

(13) $$w(x)=\frac{x^{2}}{2R}.$$

Let *x* = *l*; then, the deflection of the free end of the cantilever δ can be easily obtained. According to Equation (13), it can be found that $\delta \propto l^{2}$, which indicates that the deflection of the free end of the cantilever is proportional to the square of the beam length.

## 3. Results and Discussion

In computation, the geometrical parameters of the microcantilever are taken as: *l =* 800 μm, *b =* 100 μm, *h*_Si_ = 0.9 μm, *h*_Cr_ = 2.5 nm, *h*_Au_ = 25 nm [24]; the material parameters are taken as: *E*_Si_ = 169 GPa [9], *E*_Cr_ = 279 GPa, *E*_Au_ = 78 GPa; the other parameters: *a* = 0.22 nm [22], *k*_B_ = 1.38 × 10^−23^ J K^−1^, *T =* 298 K.

### 3.1. Comparison between Predicted and Experimental Radius of Curvatures

**Figure 2:**
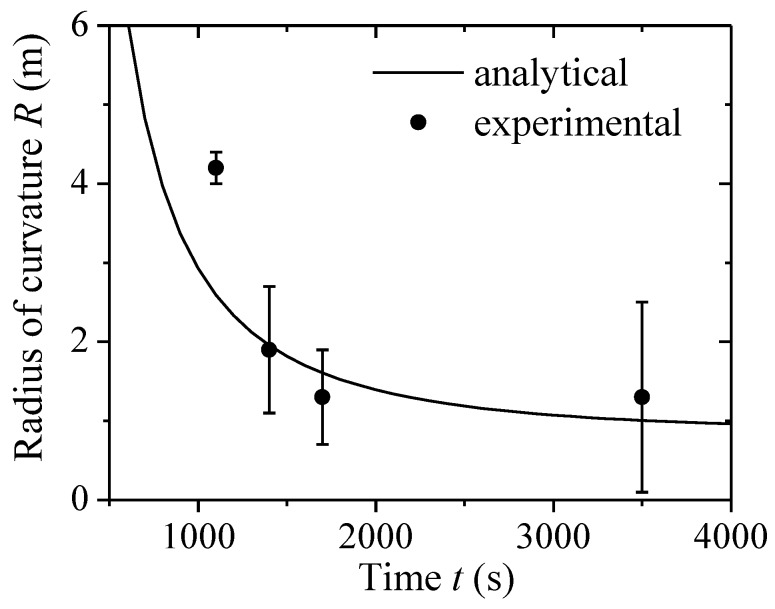
Radius of curvature of a DNA-microcantilever sensor as a function of adsorption time.

In order to illustrate the efficiency of the above method, a comparison between the predicted and experimental radius of curvature is discussed. Strictly speaking, the chemical reaction is a long-term process; it is a time-varying parametric process for real-time detections. Because DNA adsorption on one surface of the cantilever is much slower than elastic deformation, the chemical reaction is a slow, time-varying dynamical process. Therefore, the deformation of the DNA-microcantilever biosensor is treated as a static equilibrium problem. Based on the Langmuir isothermal adsorption theorem, the adsorption between analytes and the surface cantilever can be depicted [26,29,30]. The surface grafting density can be written as: *η* *=* *η*_0_(1−*e*^−^*^kt^*), where *η*_0_ is the grafting density of the DNA chain at steady state, *t* is the adsorption time, and *k* is the rate constant. Figure 2 shows the radius of curvature of a microcantilever-based DNA sensor as a function of adsorption time from the present model and Jeon’s experimental data [24] when *N =* 20 nt, *η*_0_ = 0.05 chain nm^−2^, *I =* 1 M, and *k =* 0.8 × 10^−^^3^ s^−1^. 

Our analytical predictions generally agreed with Jeon’s experimental data. The radius of curvature decreased with the enhancement of adsorption time. The reason is that the number of adsorbed DNA chains increased with the increase of adsorption time, which enhanced the contribution from the conformational entropy of DNA film. Thus, the bending cantilever became larger. However, at the early stages of adsorption experiments (i.e., <1000 s), it was not really in agreement with the experimental data. During the initial adsorption period, the adsorption of DNA molecules on gold surfaces was random, and the conformation of DNA molecules was a disordered monolayer with the nucleotide chain. Therefore, in such conditions, the ordered conformation assumption was no longer suitable, which resulted in the difference between the radius of curvature from theoretical predictions and experimental results in the early stages of the adsorption process.

### 3.2. Effect of Position on Deflections

**Figure 3:**
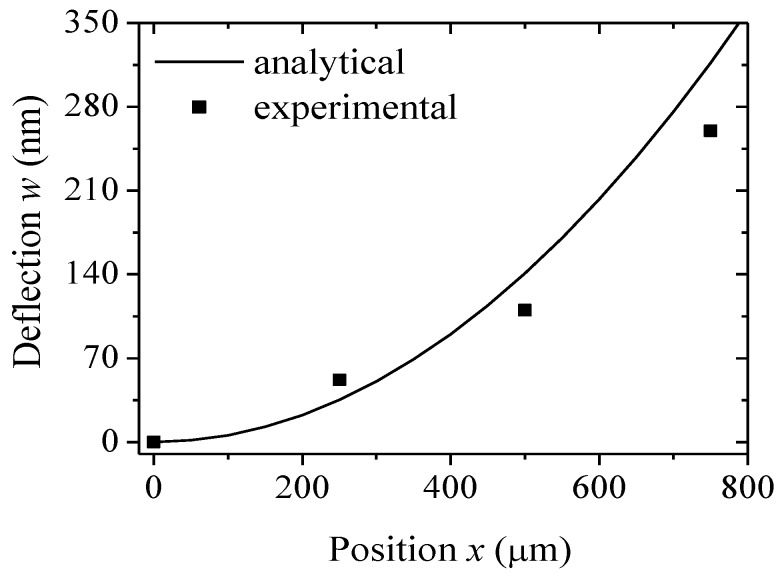
Steady deflection of a DNA-microcantilever sensor at each position of the cantilever.

To further validate this model, the steady deflection of the DNA-microcantilever sensor was predicted. For the steady deflection, no further deflection occurred, suggesting that the adsorption of thiolated DNA chains had reached an equilibrium coverage. The related parameters are the same as those in Figure 2. The predicted steady deflection of the cantilever at each position when *η* *=* *η*_0_ is illustrated in Figure 3. For comparison purposes, the experimental data by Jeon et al. [24] are also shown in Figure 3. Due to the lack of error bars of steady deflections in Jeon’s experiment, however, there are no error bars in Figure 3. It can be seen that the analytical prediction is consistent with the experimental results. The bending profile of the cantilever with a thiolated DNA chain closely follows a circular trajectory. Because the contribution of interchain interactions of the DNA chain to motion is relatively small, the Euler–Bernoulli beam theory can describe the nanomechanics of microcantilevers induced by DNA adsorption.

### 3.3. Effect of Chain Length of DNA Chain on Tip Deflections

**Figure 4:**
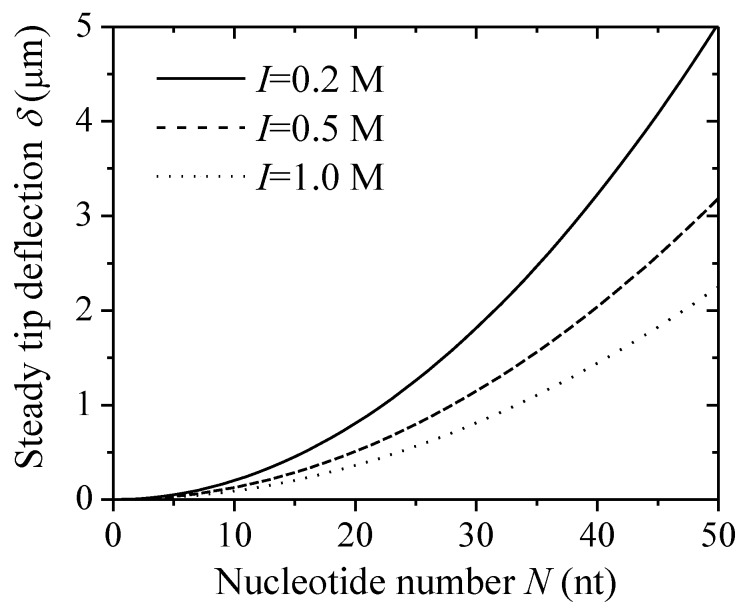
Steady tip deflection of a DNA-microcantilever sensor as a function of nucleotide number under different salt concentrations.

In the microcantilever-based system, the steady deflection of the free end of the cantilever (i.e., steady tip deflection) was detected to analyze the corresponding surface loadings (i.e., surface stress) [13,16]. Experiments have shown that the steady tip deflection of a DNA-microcantilever sensor can be induced by many factors. Here, Figure 4 shows the changes in steady tip deflections with a nucleotide number at given salt concentrations (*η* *=* 0.05 chain nm^−2^). The steady tip deflections are a strong function of the nucleotide number, and scale with *N*^2^. This result is in quantitative agreement with that from the Monte Carlo (MC) simulations [21]. In addition, with the increase of salt concentration, the steady tip deflection decreases. This reduces the relative contribution of the excluded-volume energy of DNA film to nanomechanical motion.

### 3.4. Effect of Thickness of Substrate on Tip Deflections

**Figure 5:**
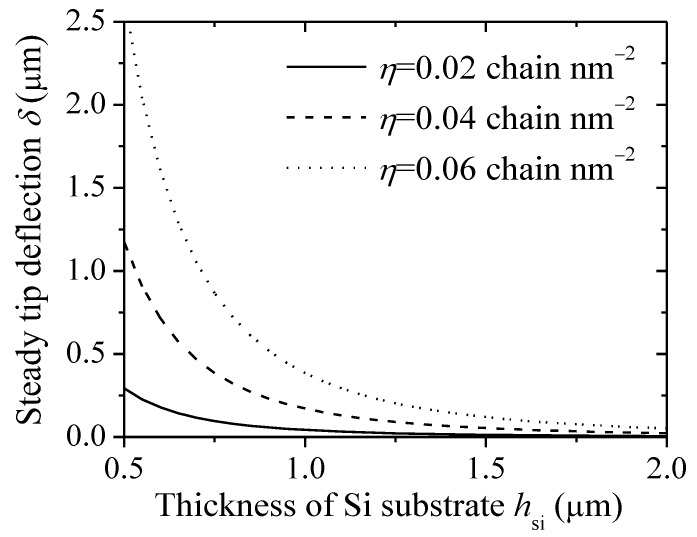
Steady tip deflection of a DNA-microcantilever sensor as a function of thickness of Si substrate under different grafting densities.

For improving the sensitivity and reliability of the microcantilever-based system, an approach of changing the substrate properties has been developed [14,15]. The effect of the thickness of Si substrate on deflection is discussed. Figure 5 shows the steady tip deflection of a microcantilever-based DNA sensor as a function of the thickness of Si substrate under different grafting densities (*N =* 20 nt, *I =* 1 M). The steady tip deflection decreases with the increase of Si thickness due to high bending stiffness values. Therefore, in order to enhance the deflection signal, it is desirable to make the cantilever as thin as possible. Additionally, the steady tip deflection increases as the grafting density increases. The reason is that the contribution of conformational free energy of DNA film enhances, which causes the increase of the steady tip deflection.

### 3.5. Effect of Elastic Modulus of Substrate on Tip Deflections

**Figure 6:**
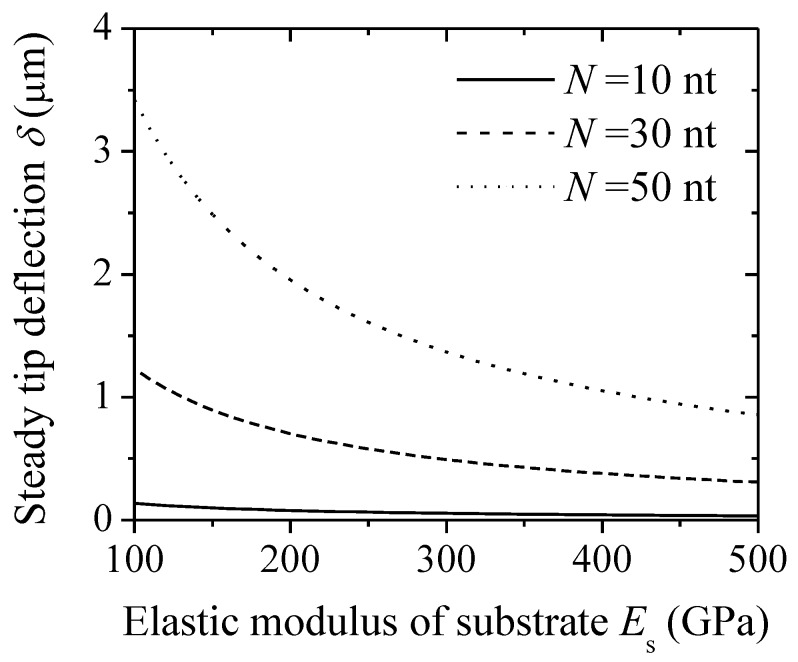
Steady tip deflection of a DNA-microcantilever sensor as a function of the elastic modulus of a substrate under different nucleotide numbers.

In addition to the effect of the thickness of substrate on tip deflections, the elastic modulus of substrate also affects deflections. Figure 6 shows the steady tip deflection of a microcantilever-based DNA sensor as a function of the elastic modulus of substrate *E*_s_ under different nucleotide numbers (*η* = 0.05 chain nm^−2^, *I* = 1 M). From the figure, the steady tip deflection decreases with the increase of the elastic modulus of substrate at a given nucleotide number. As the elastic modulus of substrate increases, the total bending stiffness for multilayer beams enhances, which decreases the steady tip deflection. It can be inferred that a cantilever with a lower elastic modulus will produce a greater deflection signal, such as polydimethylsiloxane (PDMS) [31,32]. Additionally, the steady tip deflection increases as the nucleotide number increases.

## 4. Conclusions

An energy-based model is presented to understand the bending deformation of microcantilevers induced by ssDNA adsorption. The radius of curvature and deflection of the cantilever were determined by minimizing the total free energy of the DNA-microcantilever sensor, which included the excluded-volume energy and the polymer stretching energy of DNA film, and the mechanical energy of three non-biological layers. The efficiency of the present model was confirmed through comparison with Jeon’s experimental data. The predicted results showed that DNA adsorption can significantly induce deflections of the cantilever, which depends not only on the length and grafting density of DNA chain, but also on the salt solution concentration. It was also revealed that the thickness and elastic modulus of substrate have significant effects on the deflections. This study will help to create an optimal design of microcantilever-based biosensors. It should be noted that the assumption of the ordered conformation of DNA adsorptions is unsuitable during the initial adsorption period. In addition, some physical properties (pH, temperature, etc.) of the medium can affect the deflections of the biosensors. The studies of the effects of the random adsorption and other physical properties of the medium on deflections are worth investigating in the future.
